# Metabolomic and transcriptomic analysis of the flavonoid biosynthesis pathway in *Epimedium sagittatum* (Sieb. et Zucc.) Maxim. from distinct locations

**DOI:** 10.3389/fpls.2024.1424956

**Published:** 2024-06-11

**Authors:** Shuyun Tian, Xingbin Lv, Mengxue Li, Qin Tang, Huilian Huang, Shengfu Hu, Fengqin Li, Yanqin Xu

**Affiliations:** College of Pharmacy, Jiangxi University of Chinese Medicine, Nanchang, China

**Keywords:** *Epimedium sagittatum* (Sieb. et Zucc.) Maxim, medicinal plant resource, flavonoid biosynthesis, gene expression, diversity

## Abstract

*Epimedium sagittatum* (Sieb. et Zucc.) Maxim. (ESM) which accumulates several principal flavonoid compounds including epimedin A, B, C and icariin, is extensively utilized in traditional herbs for sexual dysfunction, osteoporosis etc. In China, ESM has a wealth of wild plant resources and characterized by significant variability in medicinal compounds accumulation. Understanding the diversity of ESMs can lead to better utilization of these plant resources. In this study, we integrated the metabolomic and transcriptomic analysis of three ESMs that originated in Anhui, Hubei and Jiangxi in China. Results showed that the flavonoid biosynthesis as well as the related gene expression in these ESMs revealed substantial differences. For example, the epimedin A, B, C and icariin as well as some related gene expression in ESMs from Anhui are significantly lower than those of in others. These results suggested that the ESMs from wild population without quality checkout may not be suitable for directly use as the materials for preparation of Chinese medicine and ESMs with different accumulation of metabolites could be used for distinct applications.

## Introduction

*Epimedium sagittatum* (Sieb. et Zucc.) Maxim (ESM), as one of the *Epimedii Folium* (named as Yin-yang-huo in Chinese) in the Chinese Pharmacopoeia (Pharmacopoeia of the People’s Republic of China, 2020), contains specific medicinal flavonoid compounds. It is extensively utilized in traditional Chinese medicine for treating sexual dysfunction (Jiang et al., 2016), osteoporosis (Shi et al., 2022), tumors (Li et al., 2015) and cardiovascular disease (Zeng et al., 2022). Notably, the Icaritin Soft Capsule, derived from Epimedii Folium, was launched as a pioneering new drug in China in 2022 (The National Medical Products Administration of China, 2022), yielding significant economic benefits.

To date, over 300 compounds have been identified within the *Epimedium* genus, including four specific compounds: epimedin A, B, C and icariin (Ren et al., 2018), which serve as bioactive markers for *Epimedium*’s quality control (Xie et al., 2010; Ma et al., 2011). China has abundant wild resources of *Epimedium*, with considerable variation in the levels of these four compounds, impacting the raw material’s final quality (Li et al., 2021). Although several studies have been reported the accumulation of these main compounds in *Epimedium*, there are still lacking reports for some populations of wild *Epimedium*, such the ESMs. 4

**Figure 1:**
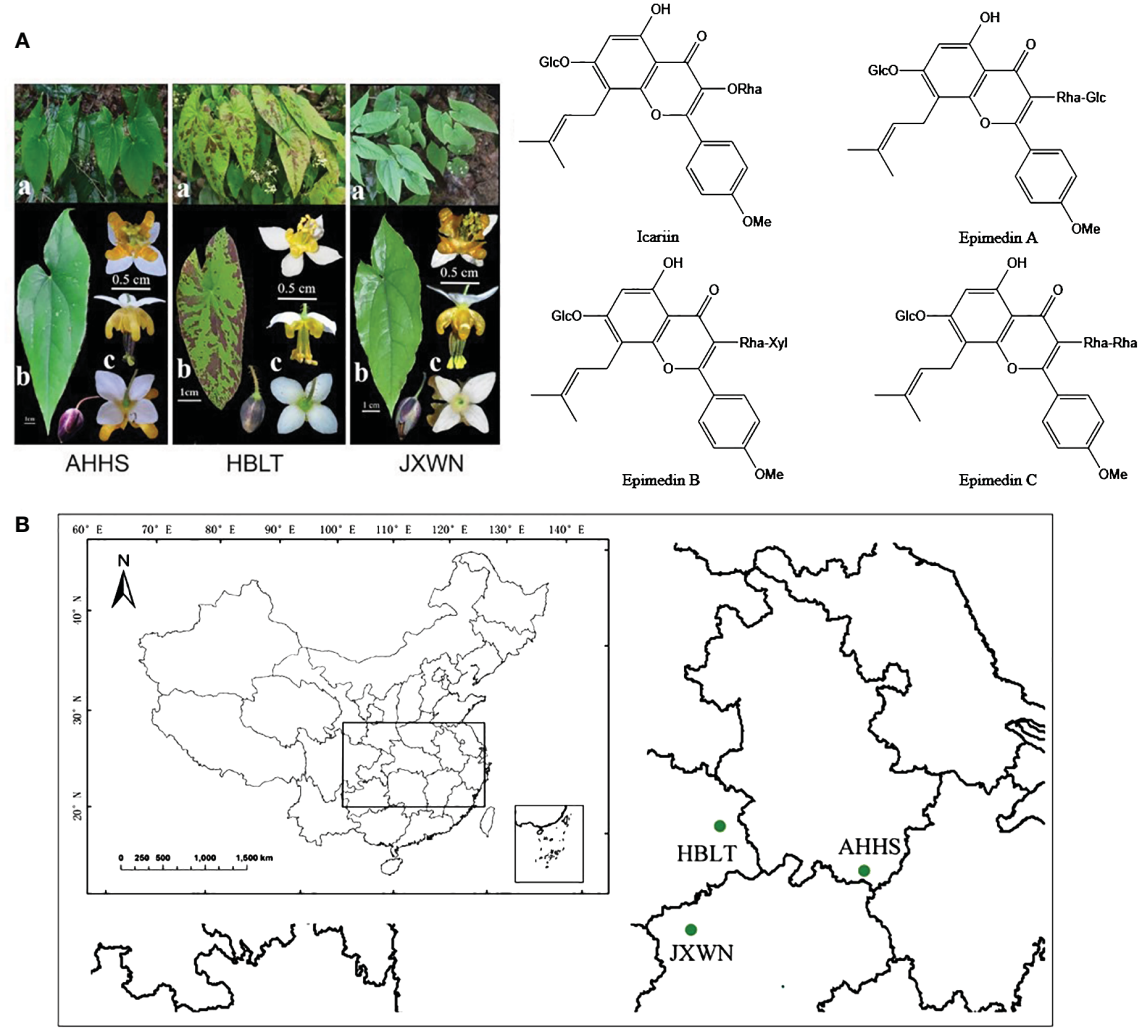
The resource of ESMs in China. **(A)** The phenotype of three ESMs (left panel) and main flavonoids compounds (right panel) of ESM. a, whole plants of three ESMs; b, leaf; c, flower; **(B)** The collecting locations of three ESMs including HBLT, AHHS, JXWN in China.

Here, we conducted an integrated metabolomic and transcriptomic analysis of three ESMs from Anhui, Hubei, and Jiangxi, China (**Figures 1A, B**). This comprehensive analysis revealed distinct metabolomic and gene expression profiles among the three ESMs, expanding our understanding of metabolites accumulation in ESMs and supporting their further potential applications in Chinese medicine preparation.

## Materials and methods

### Plant materials

Three-year-old plants of ESMs were collected from Anhui (Tangkou Town, Huangshan City, N 118° 10′ 21.3”, E 30°06′ 3.72”), Hubei (Sanlifan Town, Huanggang city, N 115° 17′ 59.88”, E 30° 54′ 21.18”) and Jiangxi (Songxi Town, Jiujiang City, N 115° 02′ 0.72”, E 29°19′ 37.38”) provinces in 2012, and then were grown in the green garden for *Epimedium* cultivation (N 28° 40′ 33.8″, E 115° 44′ 21.4″) at Jiangxi University of Chinese Medicine for nine years to stabilize the active components *in vivo*. Mature trifoliolate leaves (**Figure 1A**) from three ESMs were collected and flash-frozen in liquid nitrogen, freeze-dried under vacuum and stored at -80 °C for subsequent experiments.

### Metabolite extraction

Mature trifoliolate leaves from three ESMs were sampled and lyophilized in a vacuum freeze-dryer (Scientz-100F) and ground into powder with zirconia beads (MM 400, Retsch) for 1.5 min at 30 Hz. Then, 100 mg of powders were suspended in 1.2 mL of 70% aqueous methanol solution. The solution was vortexed for 30 minutes and placed in a refrigerator at 4°C overnight for metabolite extraction. Then, the samples were centrifuged at 12,000 rpm for 10 min and the supernatants were filtered with the 0.22 μm filter (SCAA-104, ANPEL) before UPLC-MS/MS analysis.

### UPLC-MS/MS analysis

The extracts were analyzed using a UPLC-ESI-MS/MS system (UPLC, SHIMADZU Nexera X2; MS, Applied Biosystems 4500 Q TRAP) equipped with a Agilent SB-C18 UPLC column (1.8 µm, 2.1 mm × 100 mm). Mobile phase A consisted of 0.1% formic acid in acetonitrile, while mobile phase B consisted of 0.1% formic acid in water. The column was eluted in the running program at a flow rate 0.35 ml/min: 95: 5 (v/v) at 0 min, 5:95 (v/v) at 9.0 min, 5:95 v/v at 10.00 - 11.10 min, 95:5 v/v at 14 min. The elution was then detected by an ESI-triple quadrupole-linear ion trap (QTRAP) - MS (AB4500 Q TRAP UPLC/MS/MS System) which equipped with an ESI Turbo Ion-Spray interface. The ESI source operation parameters were set as followed: turbo spray ion source; source temperature 550°C; ion spray voltage (IS) 5500 V (positive ion mode) - 4500 V (negative ion mode); the pressure of ion source gas I (GSI), gas II (GSII), curtain gas (CUR) was set at 50, 60, and 25.0 psi, respectively; the collision-activated dissociation (CAD) was set to high. Instrument tuning and mass calibration were performed with 10 and 100 μmol/L polypropylene glycol solutions in QQQ and LIT modes, respectively. QQQ scans were acquired in MRM experiments with collision gas (nitrogen) set to medium. DP and CE for individual MRM transitions were done with further DP and CE optimization. A specific set of MRM transitions were monitored for each period according to the metabolites eluted within this period.

Qualitative analysis of primary and secondary MS data was conducted using the self-compiled database MWDB (MetWare biological science and Technology Co., Ltd., Wuhan, China). Repeated signals of K^+^, Na^+^, NH4^+^, and other small molecular weight compounds were eliminated during identification. The quantitative analysis of metabolites were based on the MRM mode. The characteristic ions of each metabolite were screened through the QQQ mass spectrometer to obtain the signal strengths. The mass spectrometry data were processed using Analyst 1.6.3 software (AB SCIEX, Ontario, Canada). Metabolites with VIP ≥1, |log_2_ (fold change)| ≥ 1 and *p* < 0.05 were considered as the differential metabolites.

### Transcriptome library preparation

Mature trifoliolate leaves were sampled and mixed for total RNA extraction using the RNAprep Pure Plant Kit (Tiangen, China) following the manufacturer’s instructions. The mRNA was pulled down using 5 μg total RNA using the magnetic Dynabeads Oligo (dT)_25_ (Invitrogen, USA). The transcriptome libraries were prepared using the VAHTS Universal DNA Library Prep for Illumina V2 Kit (Vazyme, China) and sent to Illumina HiSeq 3000 for sequencing in paired-end mode (PE150).

### Transcriptome *de novo* assembly, gene annotation, expression calculation and differentially analysis

The raw data of RNA-seq were firstly trimmed to obtain clean reads with high sequencing quality (Phred score ≥ 30) and length longer than 50 bases with Trim Galore software (https://github.com/FelixKrueger/TrimGalore). The clean data were then aligned to the plant rRNA sequences database (https://www.plantrdnadatabase.com) for removing rRNA reads. The filtered RNA-seq reads were merged for *de novo* assembly using the Trinity software with min_kmer_cov set to 4 and other parameters in default values (https://github.com/trinityrnaseq). TransDecoder software was employed for the prediction of open reading frames (ORFs) (https://github.com/TransDecoder) and CD-Hit (https://sites.google.com/view/cd-hit) was used to remove the redundancy of protein sequences by the parameters set as “-c 0.8 -n 2”. For the gene annotation, the predicted protein sequences were submitted to the EGGNOG-mapper (http://eggnog-mapper.embl.de/) by the default parameter for GO, KEGG annotation etc. The transcription factors were identified by the iTAK pipeline (http://itak.feilab.net/cgi-bin/itak/index.cgi). Salmon software was employed to measure to expression level (TPM, Transcripts Per Kilobase of exonmodel per Million mapped reads) for each unigenes and DEseq2, a R package was used for identification of the differentially expression genes (DEGs). The DEGs with the fold change > 2 and the FDR < 0.05 were further extracted for the downstream analysis.

### Quantitative PCR

Total RNA was isolated from the mature trifoliolate leaves of ESMs using the UNlQ-10 column TRIzol Total RNA Extraction Kit (Sangon Biotech), following the manufacturer’s instructions. The integrity and concentration of the RNA were assessed via gel electrophoresis and quantification with an SMA 4000 microspectrophotometer (Merinton Instrument). Subsequently, cDNA synthesis was conducted using 1 µg of total RNA and the Maxima Reverse Transcriptase (Thermo Scientific). Quantitative PCR (qPCR) analyses were performed with the SYBR Premix ExTaq Mix (Takara, Japan) on a LightCycler 480 II Real-Time PCR System (Roche), employing a thermal cycling protocol of an initial denaturation at 95°C for 3 min, followed by 45 cycles of 95°C for 15 s and 60°C for 30 s. The relative gene expression level was normalized to an internal control, *EsActin* (Huang et al., 2015). Primers used are listed in **Supplementary Table S1**.

## Results

### Metabolic differences of three ESMs

In this study, we focused on three wild ESMs (**Figure 1A**) collected from Anhui, Jiangxi, and Hubei provinces, which were subsequently named as AHHS, JXWN, and HBLT (**Figure 1B**), respectively. To compare the metabolite profiles among AHHS, JXWN, and HBLT, leaf samples were analyzed using UPLC-MS/MS based on the total ion chromatogram (TIC outline) (**Supplementary Figure S1**).

**Figure 2:**
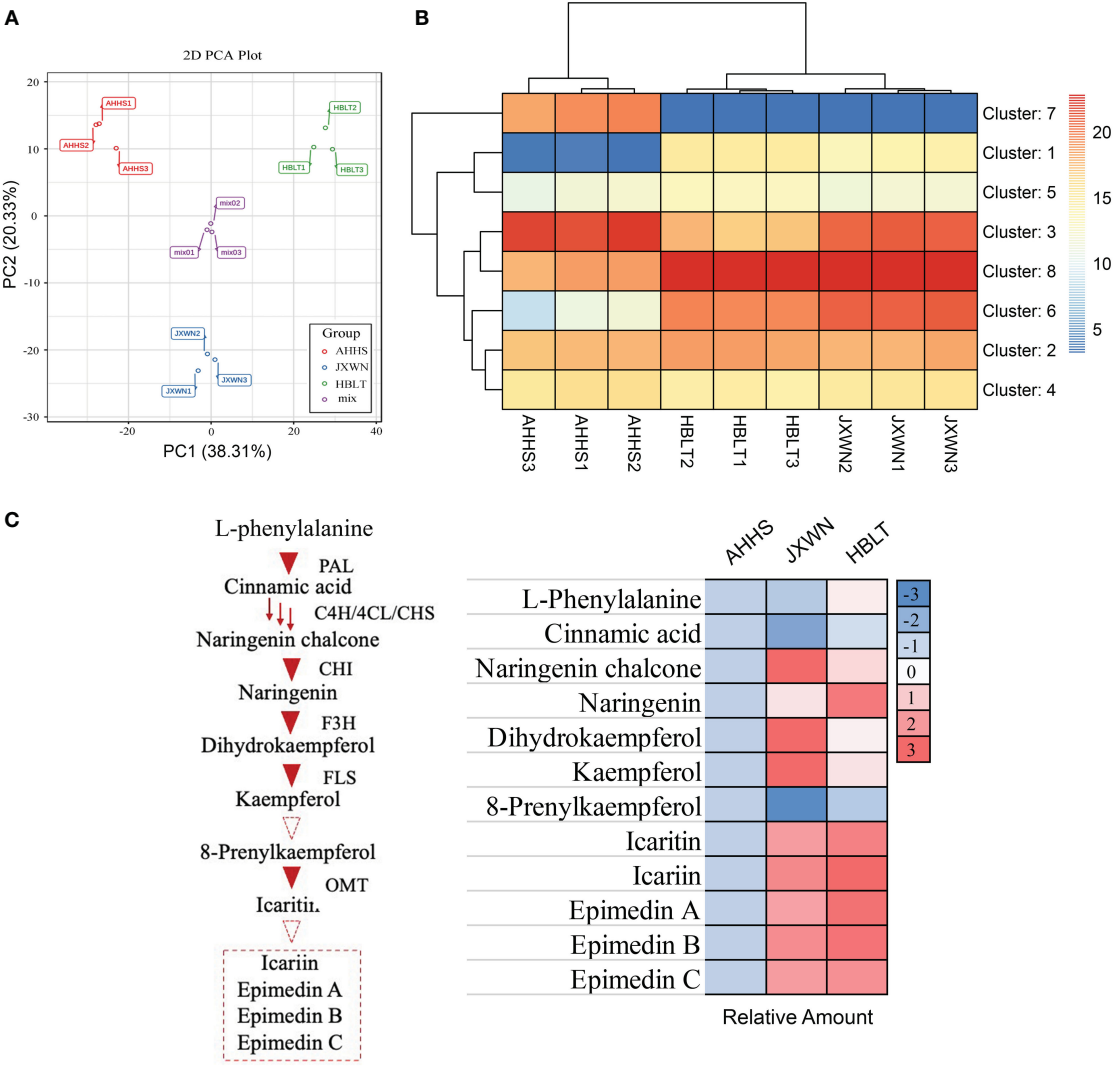
The measurement of metabolites in three ESMs including AHHS, JXWN and HBLT. **(A)** Principal component analysis (PCA) of the flavonoids compounds among AHHS, JXWN and HBLT. **(B)** Heatmap showed the eight clusters (by K-mean algorithm) of these flavonoids compounds among AHHS, JXWN and HBLT. **(C)** Twelve flavonoids compounds related to the Icariin or Epimedin biosynthesis was selected to showed the different level among AHHS, JXWN and HBLT. Left panel, the deduced biosynthesis pathway of Icariin or Epimedins. The related genes also showed besides the triangles. Right panel, the relative amount of these twelve flavonoids compounds among AHHS, JXWN and HBLT. The mean amount was normalized to that of AHHS.

In total, thousands of metabolites were identified, including 151 flavonoids that were the focus of this study (**Supplementary Table S2**). Principal component analysis (PCA) revealed that 58.64% of the total variance among the samples could be attributed to PC1 (38.31%) and PC2 (20.33%) (**Figure 2A**), demonstrating significant variation in flavonoid compositions among the leaf samples from AHHS, JXWN, and HBLT.

Additionally, hierarchical cluster analysis (HCA) was performed on these metabolites. Generally, the 151 flavonoid-related compounds were categorized into eight clusters (**Figure 2B****;** **Supplementary Table S3**). Flavonoid compounds in Cluster 7 were more abundant in AHHS compared to HBLT and JXWN, whereas those in Cluster 1 were less abundant in AHHS. We also examined twelve compounds associated with the biosynthesis of icariin and epimedins A, B, C, and the result indicated that most of these compounds, such as the epimedins, were present at higher levels in JXWN and HBLT than in AHHS (**Figure 2C**).

These findings suggested significant differences in flavonoid profiles among these ESMs from different locations. Notably, ESM resources like AHHS, which contain lower levels of icariin and epimedins A, B, C, may not be directly suitable for medical preparation.

### *De novo* transcriptome assembly of ESMs

Although the complete biosynthetic pathways of icariin and epimedins A, B, C in ESMs remain to be elucidated, as indicated in **Figure 2C**, several genes implicated in flavonoid biosynthesis have been identified. These genes provide a partial representation of the gene expression profiles across the three ESM s. Consequently, we conducted a transcriptomic analysis of these ESMs. Nine RNA-seq libraries were constructed from leaf samples of AHHS, JXWN, and HBLT (three biological replicates for each). Approximately 43.8 to 52.7 million raw reads were generated for each library (**Supplementary Table S4**).

Given the absence of a reference genome for ESM, we employed a *de novo* transcriptomic assembly approach to identify the full-length sequences of flavonoid-related genes. Initially, low-quality reads (base accuracy below 99.9%) and reads shorter than 50 bp were discarded. Subsequently, rRNA sequences were removed from the filtered reads by alignment to plant rRNA sequences from the Plant rDNA Database. The remaining reads were then assembled using the Trinity software (Haas et al., 2013). The assembled unigenes were analyzed for open reading frame (ORF) prediction using TransDecoder, and proteins shorter than 150 amino acids were excluded, resulting in 68,413 unigenes. These unigenes were further deduplicated using the CD-Hit program, yielding 31,945 non-redundant unigenes for further analysis (**Supplementary Table S5**). The contig N50 value, average contig length, and GC content of these unigenes were 1,344 bp, 1,081.2 bp, and 44.06%, respectively.

**Figure 3:**
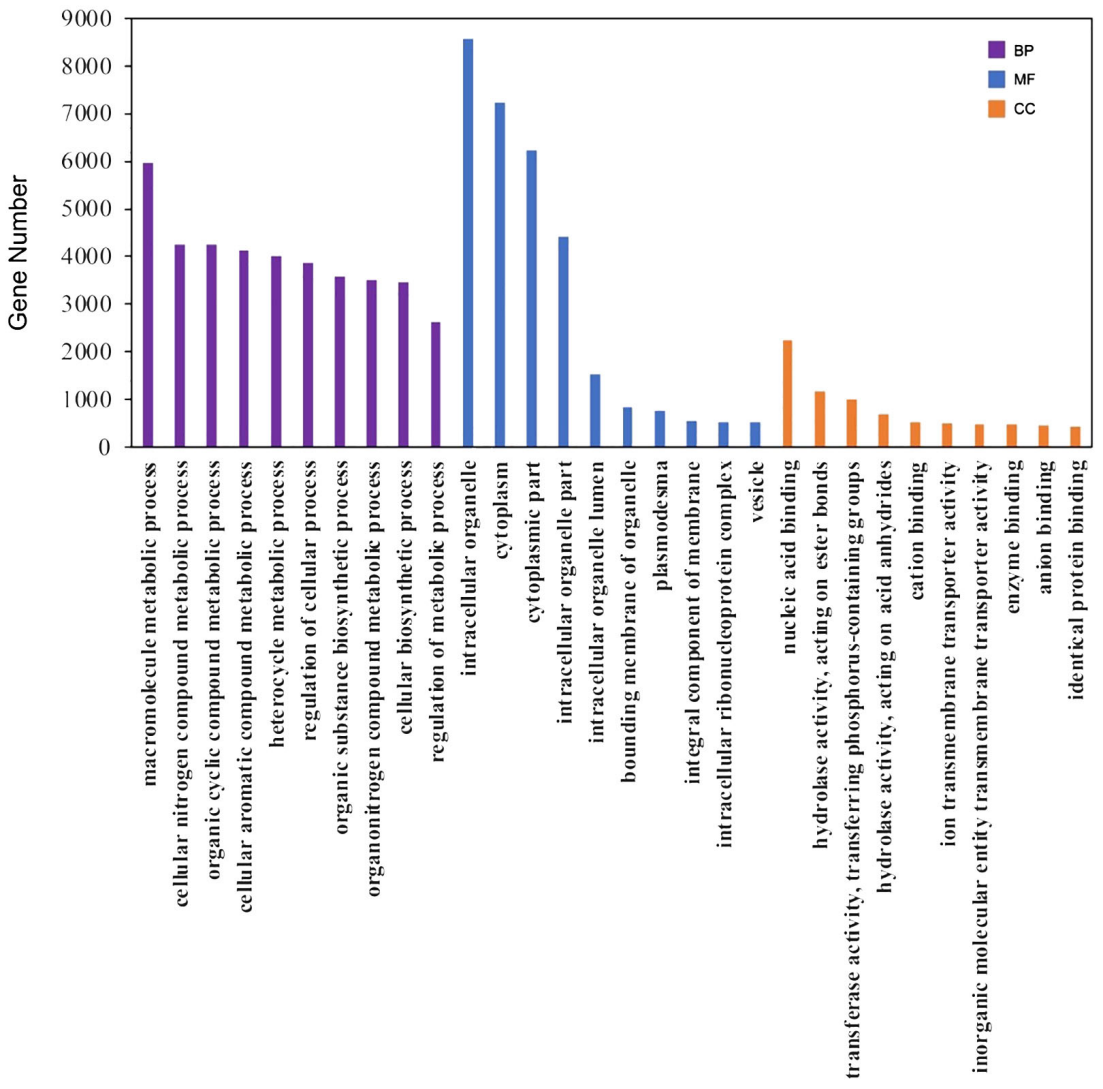
GO annotation of the unigenes. The top 10 ranked GO terms in the biological process (BP), molecular function (MF) and cellular component (CC) categories were shown as the examples here.

For annotation, the protein sequences of the 31,945 unigenes were aligned to multiple databases, including COG, Pfam, GO, and KEGG, using the EGGNOG-mapper pipeline. A total of 24,322 unigenes (76.14%) were successfully annotated (**Supplementary Table S6**). Specifically, 22,960 (71.9%), 18,061 (56.5%), and 7,746 (24.2%) unigenes were annotated in the COG, Pfam, and KEGG pathway databases, respectively. Additionally, 12,938 unigenes (40.5%) were categorized into the three principal GO categories, including Molecular Function, Cellular Component, and Biological Process (**Figure 3**; **Supplementary Table S6**). Moreover, 1,231 unigenes (3.9%) were predicted to be transcription factors using the iTAK pipeline and were classified into 65 families (**Supplementary Table S7**). The five largest transcription factor families were bHLH, AP2/ERF, MYB, C2H2, and C3H, with approximately 68 to 88 unigenes in each category, which is consistent with the distribution observed in other plant species according to the Plant TFDB database (Guo et al., 2008).

### Identified differentially expressed genes related to flavonoid biosynthesis

**Figure 4:**
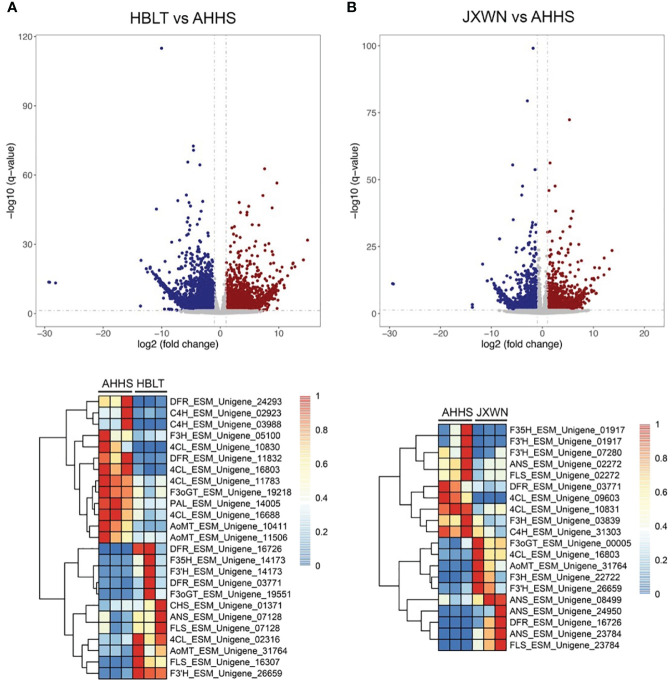
Identification of the differentially expressed genes (DEGs) among the ESMs. **(A)** Upper panel: The DEGs identified by comparing HBLT to AHHS; Lower panel: Heatmap showed the TPM of DEGs related to the flavonoid biosynthesis. **(B)** The DEGs identified by comparing JXWN to AHHS. Lower panel: Heatmap showed TPM of the DEGs related to the flavonoid biosynthesis. DEGs was defined as the genes with two-fold change and the FDR < 0.05.

Observations revealed a unique accumulation pattern of flavonoid compounds across three ESMs. This prompted an investigation into the expression of genes associated with flavonoid biosynthesis within these ESMs. To delineate differentially expressed genes (DEGs), we initially quantified the Transcripts per Million (TPM) for each unigene using the Salmon software (Patro et al., 2017). Subsequently, DEGs (HBLT vs. AHHS and JXWN vs. AHHS) were identified employing the DEseq2 program (Love et al., 2014). A comprehensive analysis yielded 7,321 DEGs in HBLT relative to AHHS, comprising 3,942 down-regulated and 3,379 up-regulated genes. In contrast, JXWN exhibited 4,504 DEGs compared to AHHS, with 1,771 down-regulated and 2,733 up-regulated genes (**Figures 4A, B****;** **Supplementary Table S8**). Notably, genes pivotal to flavonoid biosynthesis, including *phenylalanine ammonia-lyase* (*PAL*), *cinnamate 4-hydroxylase* (*C4H*), *4-coumarat: CoA ligases* (*4CL*), *chalcone synthase* (*CHS*), *flavanone 3-hydroxylase* (*F3H*), and *anthocyanin O-methyltransferase* (*OMT*), demonstrated differential expression in HBLT or JXWN relative to AHHS. This differential gene expression correlates with the distinct flavonoid metabolite profiles observed among the three ESMs.

### Co-expression analysis to identify potential TFs involved in regulation of DEGs

**Figure 5:**
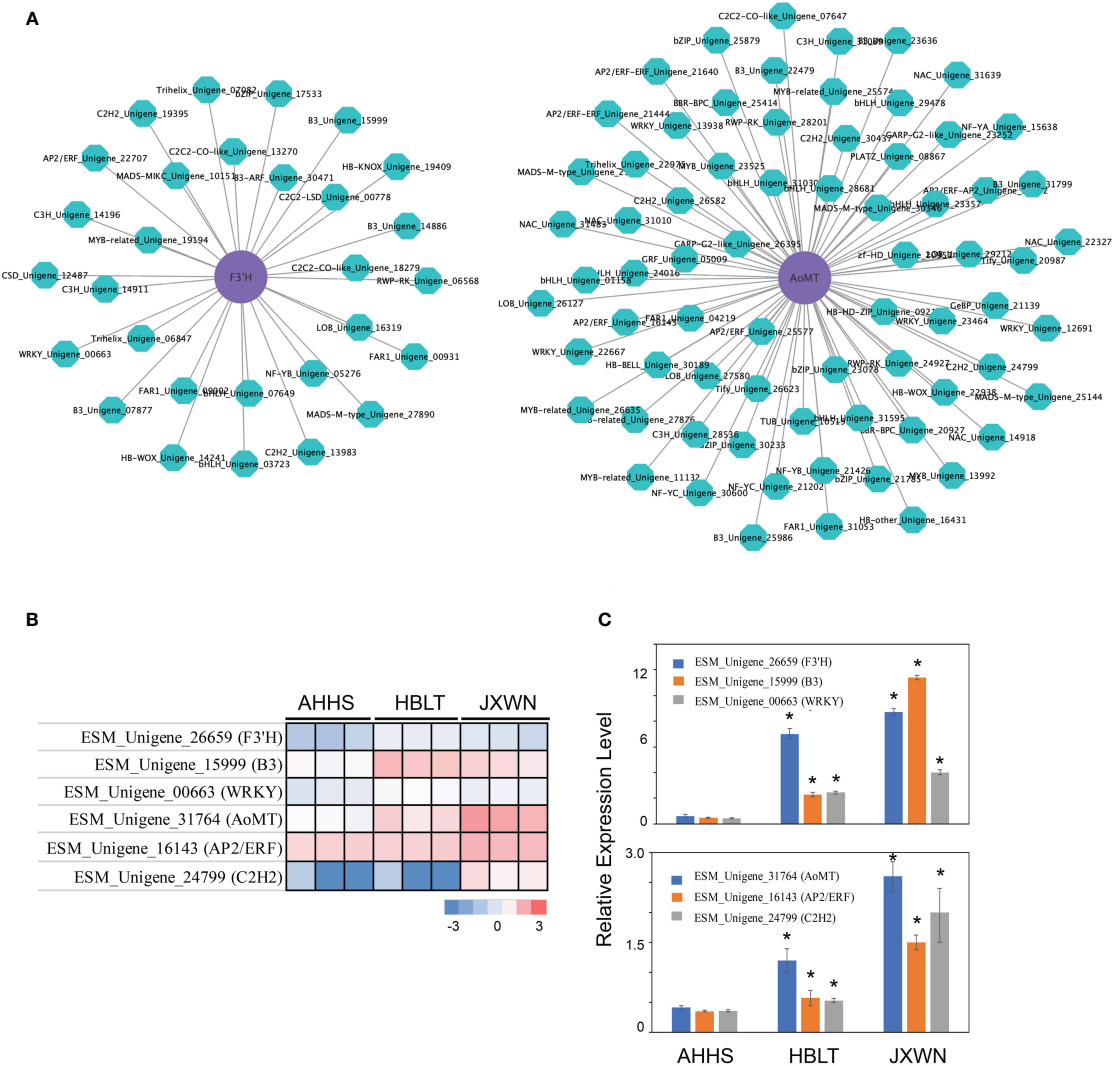
Identification of the potential key TFs involved in regulation of differentially expressed flavonoid biosynthesis related genes. **(A)** The co-expression network between the TF and the flavonoid biosynthesis related genes. The *AoMT* and *F3’H* (purple cycle) were used as the examples to show the potential regulators (green cycle). **(B)** The expression level of *AoMT*, *F3’H* as well as their potential regulators were validated by the qPCR. The heatmap (left panel) showed the TPM from RNA-seq of each gene. The qPCR validation of each gene were showed in right panel. **(C)** It represents the validation results of AoMT, F3’H, and their potential regulators via qPCR. “*” indicated the significant differences observed in HBLT, JXWN compared to AHHS (p < 0.05, by student's test).

Transcription factors (TFs) play a crucial role in regulating flavonoid biosynthesis, as previous studies (Ravaglia et al., 2013; Wang et al., 2018). To elucidate the TFs potentially regulating the differentially expressed genes (DEGs) associated with flavonoid biosynthesis, we conducted a co-expression analysis, by calculating the Pearson correlation coefficient (PCC) between TFs and DEGs involved in flavonoid biosynthesis. Gene pairs exhibiting a PCC value over 0.9 and *p* value < 0.05 were considered as co-expression partners. This analysis identified 1,148 co-expressed DEG - TF pairs (**Supplementary Table S9**), suggesting a complex regulatory network influencing flavonoid biosynthesis. Notably, two genes, *F3’H* (ESM_Unigene_26659) and *AoMT* (ESM_Unigene_31764) correlated with the epimedins accumulation (**Supplementary Table S10**) and their co-expressed TFs (**Figure 5A**), significantly upregulated in HBLT and JXWN compared to AHHS (**Figure 5B**). These findings were further validated through qPCR (**Figure 5C**). This supports the hypothesis that these TFs may play an important role in the regulation of DEGs involved in flavonoid biosynthesis.

## Discussion

In this study, we collected three ESM s from distinct locations in China, including Hubei, Jiangxi and Anhui (**Figures 1A, B**). Metabonomic and transcriptomic analyses revealed that the levels of flavonoids, such as icariin and epimedin A, B, C, as well as the expression levels of related genes, varied significantly among these ESMs (**Figures 2**, **4**). Additionally, we identified several transcription factors (TFs) that may play crucial roles in the regulation of DEGs involved flavonoid biosynthesis, as suggested by the co-expression network analysis (**Figure 5**). Collectively, these findings provide a valuable data resource for ESMs.

As an important Chinese medicinal plant, the quality of raw ESM materials has garnered widespread attention (Xie et al., 2010; Ma et al., 2011). Although numerous ESM populations originate from China, our results indicate that ESMs from different locations may exhibit variations in metabolite concentrations, particularly those with medicinal properties, such as icariin and epimedin (**Figure 2**). For instance, AHHS has the lowest flavonoid content, is not suitable for direct use in the preparation of Chinese medicine or for the extraction of active compounds. These findings underscore the necessity of quantifying the medicinal activity of raw ESM materials prior to market distribution.

In this study, we observed distinct gene expression profiles among three ESMs from Hubei, Jiangxi, and Anhui, with particular attention to the AHHS from Anhui, which exhibited the lowest flavonoid levels (**Figure 2C**). Despite identifying differential gene expression in AHHS compared to the other two ESMs, the majority of flavonoid biosynthesis-related genes detected did not elucidate the precise causes of the varied metabolite accumulation (Zou et al., 2016; Liu et al., 2021; Lou et al., 2021). Two primary reasons are posited: (1) the complete biosynthetic pathways of major flavonoids such as icariin and epimedin remain elusive to date (Huang et al., 2015; Wang et al., 2021), precluding the direct detection of gene expression responsible for their biosynthesis; (2) the absence of a reference genome for ESMs hinders the examination of genomic variations among these samples. Nevertheless, the finding that AHHS containing the lowest flavonoid levels has piqued interest, potentially serving as a critical genetic resource for future studies, such as constructing a recombinant inbred line for Quantitative Trait Locus (QTL) analysis (Mauricio, 2001).Taken together, these ESMs with varying flavonoid contents represent significant plant resources for further investigation.

## Data availability statement

All the raw data in this study can be found in the China National Center for Bioinformation (https://www.cncb.ac.cn) under the BioProject accession number PRJCA024022.

## Author contributions

ST: Writing – original draft, Writing – review & editing. XL: Writing – original draft, Writing – review & editing. ML: Writing – review & editing. QT: Writing – review & editing. HH: Writing – review & editing. SH: Writing – review & editing. FL: Writing – review & editing. YX: Methodology, Supervision, Writing – review & editing.

## Supplementary material

Supplementary Figure 1 The raw TIC of mass spectrometry for three ESMs. **(A)** ion in positive mode and **(B)** anion mode. The blue, red and green lines are indicated the samples from JXWN, HBLT and AHHS.
