# Supplementary figures and images for: Metabolomic and transcriptomic analysis of the flavonoid biosynthesis pathway in Epimedium sagittatum (Sieb. et Zucc.) Maxim. from distinct locations
Source: Front Plant Sci. 2024 Jun 11;15:1424956. doi: 10.3389/fpls.2024.1424956 (PMC11196779; doi:10.3389/fpls.2024.1424956)

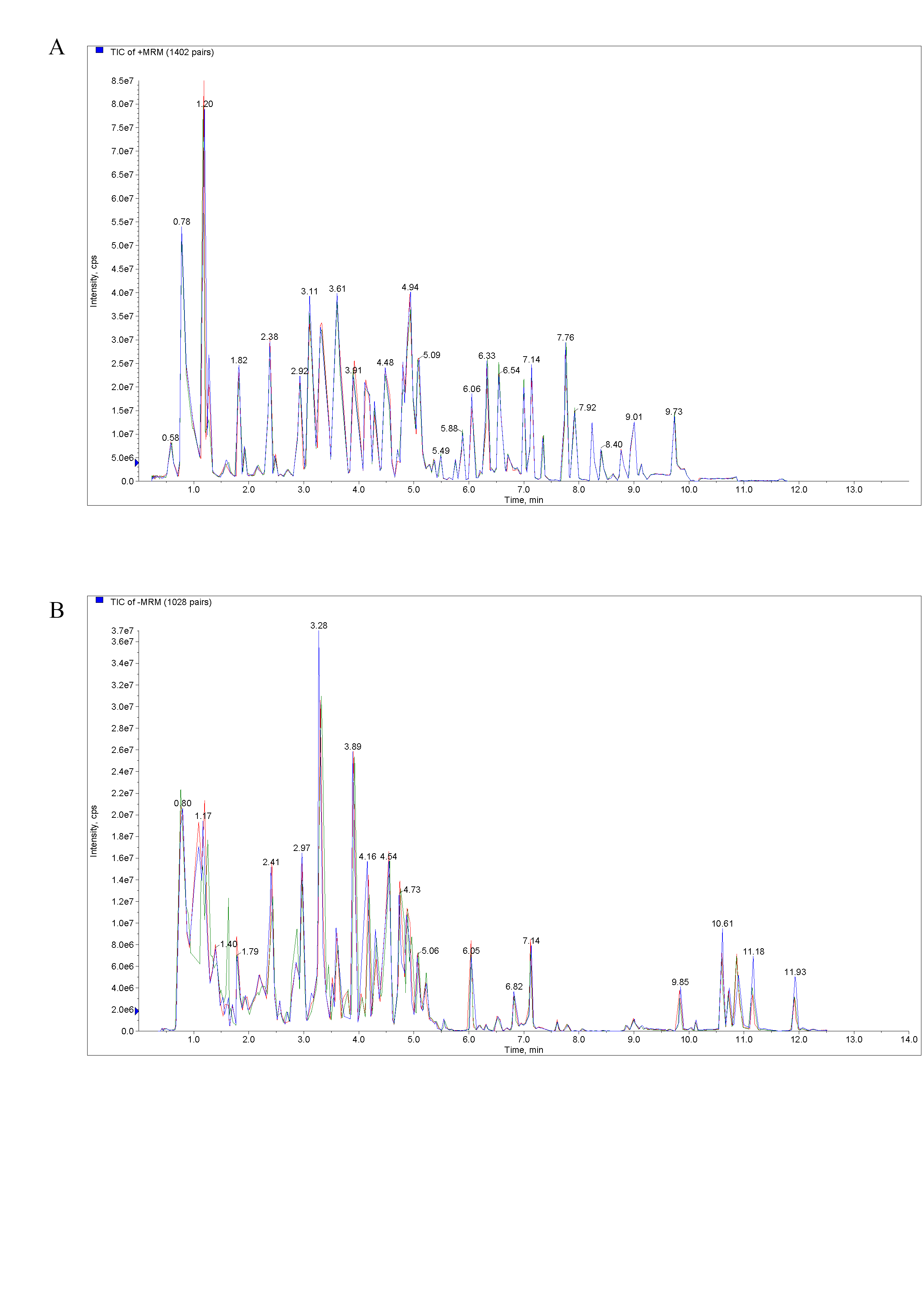

Supplement: Supplementary Figure 1 — The raw TIC of mass spectrometry for three ESMs. (A) ion in positive mode and (B) anion mode. The blue, red and green lines are indicated the samples from JXWN, HBLT and AHHS. [file Image_1.jpeg]
